# Mortality after cardiac resynchronization therapy or right ventricular pacing in transcatheter aortic valve replacement recipients

**DOI:** 10.1007/s00392-024-02450-1

**Published:** 2024-05-02

**Authors:** Johannes Kirchner, Muhammed Gerçek, Vanessa Sciacca, Jan-Christian Reil, Denise Guckel, Max Potratz, Hazem Omran, Kai Friedrichs, Thomas Eitz, Sabine Bleiziffer, Rene Schramm, Guram Imnadze, Christian Sohns, Jan Gummert, Volker Rudolph, Tanja K. Rudolph, Philipp Sommer, Thomas Fink

**Affiliations:** 1 Clinic for General and Interventional Cardiology/Angiology, Herz- und Diabeteszentrum NRW, Universitätsklinik (Ruhr-Universität Bochum), Medizinische Fakultät OWL (Universität Bielefeld), Bad Oeynhausen, Germany; 2 Clinic for Electrophysiology, Herz- und Diabeteszentrum NRW, Universitätsklinik (Ruhr-Universität Bochum), Medizinische Fakultät OWL (Universität Bielefeld), Bad Oeynhausen, Germany; 3 Clinic for Thoracic and Cardiovascular Surgery, Herz- und Diabeteszentrum NRW, Universitätsklinik (Ruhr-Universität Bochum), Medizinische Fakultät OWL (Universität Bielefeld), Bad Oeynhausen, Germany

**Keywords:** TAVR, CRT, Pacemaker, Survival, Heart failure

## Abstract

**Background:**

Permanent pacemaker implantation (PMI) is associated with increased morbidity after transcatheter aortic valve replacement (TAVR). Cardiac resynchronization-therapy (CRT) is recommended for patients if left ventricular ejection fraction (LVEF) is ≤ 40% and ventricular pacing is expected in favor to sole right ventricular (RV) pacing. Meanwhile, LVEF may recover after TAVR in patients with aortic valve disease and the benefit of CRT is unknown.

**Objective:**

To analyze the impact of CRT implantation as compared to RV pacing after TAVR.

**Methods and Results:**

Between 2012 and 2022, 4385 patients (53.1% female, mean age 81 ± 6 years) without prior PMI undergoing TAVR were retrospectively identified in our institutional registry. After stratification of patients in LVEF ≤ 40%, 41–49% and ≥ 50%, Kaplan–Meier analysis revealed significantly different survival rates in each subgroup at 5 years (37.0% vs. 43.5% vs. 55.1%; P ≤ 0.021). At multivariate regression, LVEF and new PMI after TAVR were not relevant for survival. A total of 105 patients with LVEF ≤ 40% received PMI after TAVR (86 patients with RV pacing and 19 with CRT). At 5 years, all-cause mortality was significantly lower in patients with CRT-device as compared to patients without CRT-device (Kaplan Meier estimate of 21.1% vs. 48.8%; HR 0.48, CI 0.204 – 1.128; log rank *p* = 0.045). In multivariate analysis CRT remained a significant factor for 5-year survival in these patients (HR 0.3, CI 0.095–0.951, *p* = 0.041).

**Conclusion:**

In patients undergoing TAVR, PMI did not influence 5-year survival. In patients with LVEF ≤ 40%, CRT-device implantation was associated with improved survival compared to non-CRT-device implantation.

**Graphical Abstract:**

Impact of right ventricular pacing and cardiac resynchronization therapy on patient survival in patients with transcatheter aortic valve replacement. CI = confidence interval, CRT = cardiac resynchronization therapy, HR = hazard ratio, LVEF = left ventricular ejection fraction, RV = right ventricular, TAVR = transcatheter aortic valve replacement

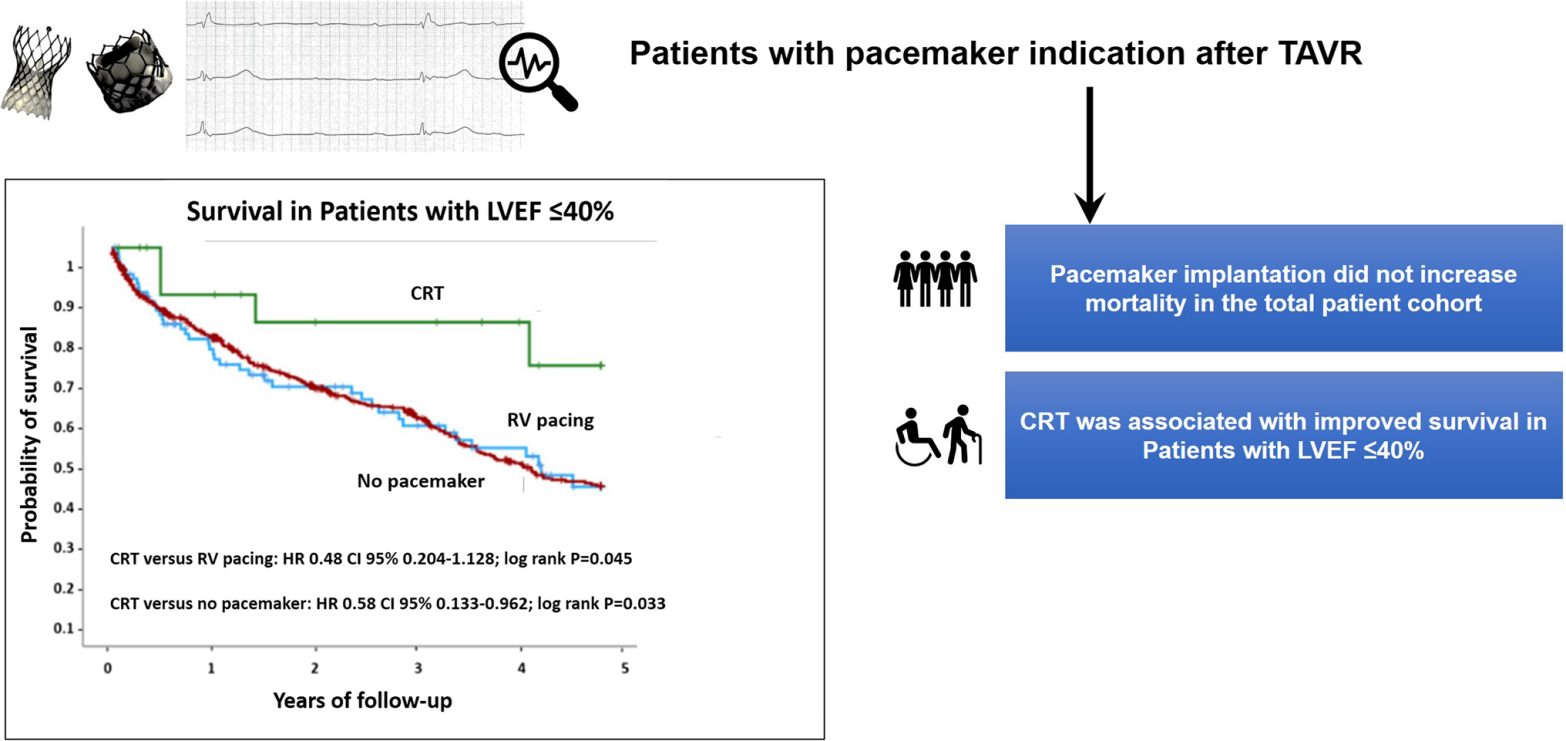

**Supplementary Information:**

The online version contains supplementary material available at 10.1007/s00392-024-02450-1.

## Introduction

After transcatheter aortic valve replacement (TAVR), new onset of conduction abnormalities occurs frequently [1]. If high grade AV-blockage persists over 24-48 h or new onset of alternating bundle branch occurs after TAVR, permanent pacemaker implantation (PMI) is recommended in current guidelines (2). Furthermore, if pre-existing right bundle branch block (RBBB) with new onset of conduction disturbance occurs, PMI is also recommended [2]. Incidence of PMI after TAVR has decreased over time but remains between 2.3% and 36.1% [3]. Data regarding the association between PMI and increased mortality and hospitalization is controversial [4–8].

In the aforementioned trials mean left ventricular ejection fraction (LVEF) was preserved in the majority of patients. Heart failure patients with a high burden of right ventricular pacing have an increased risk for hospitalization and death [9]. Hence, if LVEF is reduced and ventricular pacing is expected to occur, cardiac resynchronization therapy (CRT) is indicated [2, 10]. Current European guidelines recommend CRT implantation in patients with LVEF ≤ 40% and high-degree AV block [2] while American guidelines recommend patient evaluation for CRT in patients with clinical heart failure and LVEF 36–50% [11].

The status of CRT in patients who underwent TAVR and have an indication for PMI is unclear. Patients with severe aortic stenosis and reduced LVEF undergoing TAVR have a higher mortality as compared to individuals with preserved LVEF but that seems to be no longer significant after adjustment for clinical factors [12, 13]. Furthermore, LVEF increases frequently after TAVR and failure of LVEF improvement is associated with increased mortality [14]. Mean LVEF improvement is described to be around 13% after 1 year [14–16]. Therefore, a substantial fraction of patients with prior LVEF ≤ 40% will no longer have an indication for CRT implantation 1 year after TAVR. On the other hand, patients with PMI prior TAVR seem to have less LVEF improvement after TAVR [14]. As a consequence, there is no dedicated guideline recommendation on device selection in patients with reduced LVEF and high-degree AV block after TAVR [2, 11].

In this study we sought to analyze the impact of right ventricular (RV) pacing versus CRT on overall mortality of patients undergoing TAVR in our center.

## Material and methods

### Study design and patient selection

This single-center trial was approved by the local ethics committee and conforms with the principles outlined in the Declaration of Helsinki. Data is used from a hospital registry – a single center registry to track outcomes in patients with severe aortic stenosis undergoing TAVR at the Heart and Diabetes Centre North Rhine-Westphalia, Bad Oeynhausen, Germany. Patient selection and the required procedural technique for TAVR were decided by a heart team. Consecutive patients undergoing TAVR from 2012 to 2022 were analyzed. Patients with pre-existing cardiac implantable electronic devices were excluded from further analysis.

### Pacemaker implantation and periprocedural management

The indication for post-TAVR PMI was determined according to current guidelines (2). PMI was performed if high grade AV-blockage or alternating bundle branch with conduction disturbance occurred after TAVR. New onset of sole left bundle branch or prolonged AV-interval was not seen as indication for PMI. Patients underwent PMI during the same hospital stay as TAVR. In patients with preserved sinus rhythm, a dual-chamber device was implanted. Ventricular leads were placed at the right ventricular apex or at the interventricular septum. The mode of pacing in patients with reduced LVEF was on discretion of the treating physicians and included RV pacing as well as CRT implantation. The main reasons for CRT implantation were the individual age and clinical status of the patient as well as the expected percentage of pacing and expected LVEF recovery after TAVR which was estimated by the treating physicians. The final decision whether to implant a CRT device or to perform RV pacing was based after individual decision in each patient at the discretion of the treating physicians.

### Study outcomes

For clinical endpoints, outcomes were evaluated at hospital discharge and follow up visits if available. Patients were seen in our outpatient clinic or by the referring cardiologist after 3–6 months and then once yearly. Visits included clinical inspection and patients´ history assessment, transthoracic echocardiography, ECG and pacemaker interrogation if necessary.

Five-year outcome data were used for analysis of outcome measures. The primary endpoint was all-cause mortality after 5 years. Secondary endpoints included QRS width after PMI and LVEF improvement in patients with LVEF ≤ 40%.

For analysis we divided these patients in 3 groups based on LVEF prior to TAVR because of two reasons. First, these thresholds are cut off values for diagnosis of Heart failure with reduced ejection fraction (HFrEF = LVEF ≤ 40%), with mildly reduced ejection fraction (HFmrEF = LVEF 41–49%) and preserved ejection fraction (HFpEF = LVEF ≥ 50%). Secondly, LVEF ≤ 40% is the cut-off value for European guideline recommendation of CRT in patients with heart failure in whom a high percentage of right ventricular pacing is expected [2].

Since not all patients with LVEF ≥ 50% are diagnosed with HFpEF, this group is referred to patients with “left ventricle with preserved ejection fraction” (LVpEF). Similarly, patients with a LVEF 41–49% are referred to “left ventricle with mildly reduced ejection fraction” (LVmrEF), and those with an LVEF ≤ 40% are referred to “left ventricle with reduced ejection fraction” (LVrEF).

### Statistical analysis

Continuous parameters are reported as mean ± standard deviation (SD), whereas categorical variables are reported as frequencies and percentages. For comparison of patient characteristics and procedural data among study groups, the independent Student’s t-test, Chi-square test or Fisher-exact test was used were appropriate. A Mann–Whitney U test was used for comparison of two groups with non-parametric parameters, the Kruskal–Wallis test was used when more than 2 groups of non-parametric values were compared. Patients’ survival was estimated with Kaplan–Meier analysis and compared with log rank test between patient groups. Hazard ratios and 95% confidence interval (CI) were estimated using a Cox-regression analysis. A two-sided *p*-value < 0.05 was considered as statistically significant. All analyses were performed with SPSS software (IBM Corp., Armonk, NY, USA).

## Results

### Patient cohort

A total of 4385 patients (mean age 81 ± 6 years, 53.1% female) without PMI before TAVR were analyzed. Baseline characteristics stratified for LVEF are displayed in Table 1. Beside other findings, LVrEF patients were more often male, had higher STS-Score and EuroScore II and were more symptomatic as LVmrEF and LVpEF patients as assessed by NYHA classification. Furthermore, LVrEF patients displayed more comorbidities including chronic kidney disease, peripheral artery disease and diabetes mellitus. Echocardiographic values can be found in Supplemental Table 1. LVrEF patients had lower mean aortic valve gradient (37.9 ± 15.4 mmHg vs. 41.4 ± 16.8 mmHg vs. 47.1 ± 22.1 mmHg; *p* < 0.001) prior TAVR and smaller effective orifice area after TAVR compared to LVmrEF and LVpEF patients (1.74 ± 0.47 cm^2^ vs. 1.77 ± 0.44 cm^2^ vs. 1.81 ± 0.46 cm^2^; *p* < 0.010). However, despite a higher rate of trans-apical access in LVrEF and LVmrEF patients, rates of major complications were not significantly different among these groups. Time on intensive-care-unit (2 days (IQR 1–4 days) vs. 1 days (IQR 1–3 days) vs. 1 day (IQR 1–3 days); *p* < 0.010) and hospital stay (13 days (IQR 8–18 days) vs. 11 days (IQR 8–15 days) vs. 10 days (IQR 8–14 days); *p* < 0.001) were significantly longer in LVrEF patients compared to LVmrEF and LVpEF patients (Table 2). Furthermore, in-hospital-mortality was significantly different among groups with LVpEF, LVmrEF and LVrEF (3.4% vs. 3.0% vs. 1.6%; *p* = 0.001). There was no difference in post-procedural aortic regurgitation/ paravalvular leakage between the three subgroups.

**Table 1:** Baseline characteristics

Parameter	All	LVrEF	LVmrEF	LVpEF	*p*-value
*n*	4385	698	305	3382	
Male, *n* (%)	2055 (46.9)	433 (62.0)	175 (57.4)	1447 (42.8)	** < 0.001**
Age, years	81.4 ± 6.1	80.8 ± 7.3	81 ± 6	81.6 ± 5.8	0.149
BMI, kg / m^2^	27.3 ± 5.2	26.7 ± 5.2	27.5 ± 5.5	27.4 ± 5.1	**0.002**
Euroscore II	5.9 ± 6.5	12.9 ± 11.2	7.3 ± 6.3	4.7 ± 4.3	** < 0.001**
STS-Score	5.7 ± 4.6	7.9 ± 7	6.2 ± 4.5	5.2 ± 3.7	** < 0.001**
LVEF, %	51.7 ± 9.4	34 ± 6.3	45.2 ± 1.5	55.9 ± 4.3	** < 0.001**
Creatinine, mg/dl	1.5 ± 1.3	1.5 ± 1.1	1.4 ± 0.9	1.2 ± 0.7	** < 0.001**
NYHA I, *n* (%)	117 (2.7)	9 (1.3)	4 (1.3)	104 (3.1)	** < 0.010**
NYHA II, *n* (%)	1299 (29.6)	131 (18.8)	73 (23.9)	1095 (32.4)	
NYHA III, *n* (%)	2707 (61.7)	449 (64.3)	200 (65.6)	2058 (60.9)	
NYHA IV, *n* (%)	262(6.0)	109 (15.6)	28 (9.2)	125 (3.7)	
CVD, *n* (%)	642 (14.6)	86 (12.3)	42 (13.8)	514 (15.2)	0.133
PAD, *n* (%)	537 (12.2)	116 (16.6)	46 (15.1)	375 (11.1)	** < 0.001**
Hypertension, *n* (%)	3988 (91.1)	609 (87.2)	287 (94.1)	3097 (91.57)	** < 0.001**
Diabetes mellitus, *n* (%)	1289 (29.5)	250 (36.3)	117 (38.4)	927 (27.4)	** < 0.001**
Hyperlipidemia, *n* (%)	3395 (80.3)	525 (75.2)	249 (81.6)	2778 (82.1)	** < 0.001**
No CAD, *n* (%)	1861 (42.2)	241 (34.5)	103 (33.8)	1517 (44.9)	** < 0.001**
CAD, 1 vessel, *n* (%)	887 (20.2)	133 (19.1)	72 (23.6)	682 (20.2)	
CAD, 2 vessel, *n* (%)	635 (14.5)	105 (15.0)	56 (18.4)	474 (14.0)	
CAD, 3 vessel, *n* (%)	1002 (22.9)	219 (34.5)	74 (24.3)	709 (21.0)	
Prior stent implantation, *n* (%)	1449 (33.9)	271 (38.8)	122 (40)	1056 (31.2)	** < 0.001**
Atrial Fibrillation, *n* (%)	1618 (36.9)	326 (46.7)	148 (48.5)	1135 (33.6)	** < 0.001**
Prior Stroke / TIA, *n* (%)	3759 (85.7)	580 (83.1)	248 (81.3)	2931 (86.7)	**0.004**
Dialysis, *n* (%)	127 (2.8)	48 (6.9)	11 (3.6)	68 (2.0)	** < 0.001**
COPD, *n* (%)	856 (19.5)	154 (22.1)	60 (19.7)	642 (19.0)	0.174

Values are displayed as mean ± SD or frequencies (%). BMI = body mass index; CAD = coronary artery disease; COPD = chronic obstructive pulmonary disease; CVD = cervical vascular disease; NYHA = New York Heart Association; PAD = peripheral artery disease; STS = Society of Thoracic Surgery; TIA = transient ischemic attack

**Table 2:** Outcome measurements

Parameter	LVrEF	LVmrEF	LVpEF	*p*-value
In-Hospital Mortality, *n* (%)	24 (3.4)	9 (3.0)	54 (1.6)	**0.030**
Mortality after 5 years, *n* (%)	308 (44.1)	103 (33.8)	916 (27.1)	**0.001**
Days on ICU	2 (1–4)	1 (1–3)	1 (1–3)	** < 0.001**
Days in Hospital	13 (8–18)	11 (8–15)	10 (8–14)	** < 0.001**
Conversion to transapical, *n* (%)	4 (0.7)	3 (1.0)	8 (0.2)	0.534
Conversion to sternotomy, *n* (%)	5 (0.9)	1 (0.3)	28 (0.8)	
Device embolisation, *n* (%)	2 (0.4)	1 (0.3)	11 (0.3)	0.288
VARC Bleeding minor	32 (3.0)	10 (3.3)	101 (3.0)	0.680
VARC bleeding major, *n* (%)	16 (2.3)	3 (1.0)	68 (2.0)	
VARC bleeding lifethreating, *n* (%)	10 (1.4)	3 (1.0)	22 (0.7)	
VARC vessel complication minor, *n* (%)	16 (2.3)	8 (2.6)	123 (3.6)	0.720
VARC vessel complication major, *n* (%)	9 (1.3)	6 (2.0)	63 (1.9)	
VARC vessel complication failure of closing device, *n* (%)	1 (0.1)	1 (0.3)	12 (0.4)	
VARC stroke non disabing, *n* (%)	5 (0.7)	1 (0.3)	17 (0.5)	0.930
VARC stroke disabing, *n* (%)	4 (0.6)	1 (0.3)	19 (0.6)	
Access transfemoral, *n* (%)	557 (79.8)	244 (80.0)	2932 (86.7)	** < 0.001**
Access transapical, *n* (%)	126 (18.1)	59 (19.3)	394(11.6)	** < 0.001**
Access transaortic, *n* (%)	4 (0.6)	1 (0.3)	2 (0.1)	**0.006**
Access transsubclavian, *n* (%)	10 (1.4)	1 (0.3)	51 (1.5)	0.261
Pacemaker implantation after TAVR, *n* (%)	105 (15.0)	48 (15.7)	450 (13.3)	0.171

Values are displayed as mean ± SD, median (interquartile range) or frequencies (%). ICU = intensive care unit; IQR = interquartile range; LVEF = left ventricular ejection fraction; VARC = Valve Academic Research Consortium

### Impact of LV function on patient survival

**Fig. 1:**
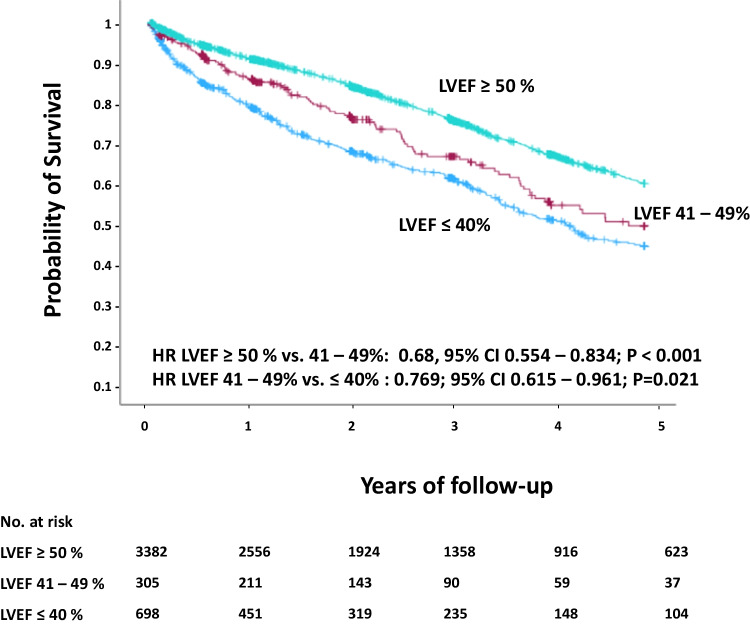
Survival after TAVR related to LVEF. CI = confidence interval, HR = hazard ratio, LVEF = left ventricular ejection fraction

After a mean follow up of 739 days (IQR 300 to 1465 days), Kaplan–Meier analysis revealed significantly different estimated survival rates in each subgroup with the lowest estimated survival after 1117 ± 30 days in LVrEF patients (37.0% vs. 43.5% vs. 55.1%; HR ≥ 50% vs. 41–49%: 0.68, CI 0.554–0.834, log rank *p* < 0.001; HR 41–49% vs. ≤ 40%: 0.769, CI 0.615–0.961, log rank *p* = 0.021) (Fig. 1). However, multivariate cox regression found age and baseline creatinine, but not LVEF as significant confounders for survival (Supplemental Table 2).

### Impact of PMI on patient survival and LV function recovery

**Fig. 2:**
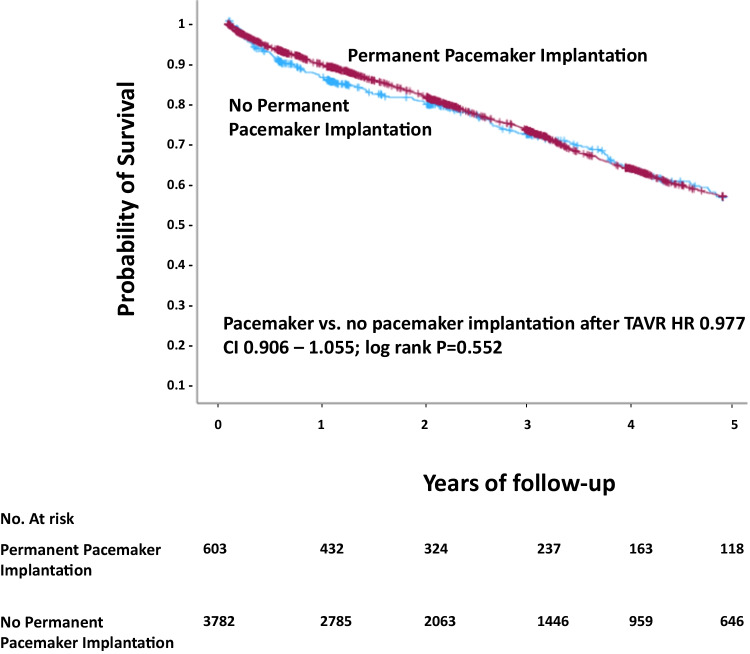
Survival after TAVR in patients with and without PMI. CI = confidence interval, HR = hazard ratio, TAVR = transcatheter aortic valve replacement

A total of 603 of 4385 patients (13.8% of all patients) underwent PMI after TAVR. There was no difference in patient survival for patients receiving PMI after TAVR as compared to patients without PMI in the overall cohort in uni- und multivariate analysis (48.6% vs. 48.5%; HR 0.977, CI 0.906–1.055; log rank *p* = 0.552) (Fig. 2, Supplemental Table 3).

Among patients receiving PMI, 105 were LVrEF patients (15.0%), 48 LVmrEF patients (15.7%) and 450 LVpEF patients (13.3%), *p* = 0.171). Of these, 19 LVrEF patients (18.1%), 1 LVmrEF patient (2.1%) and 1 LVpEF patient (0.2%) received a CRT system (*p* < 0.001).

**Fig. 3:**
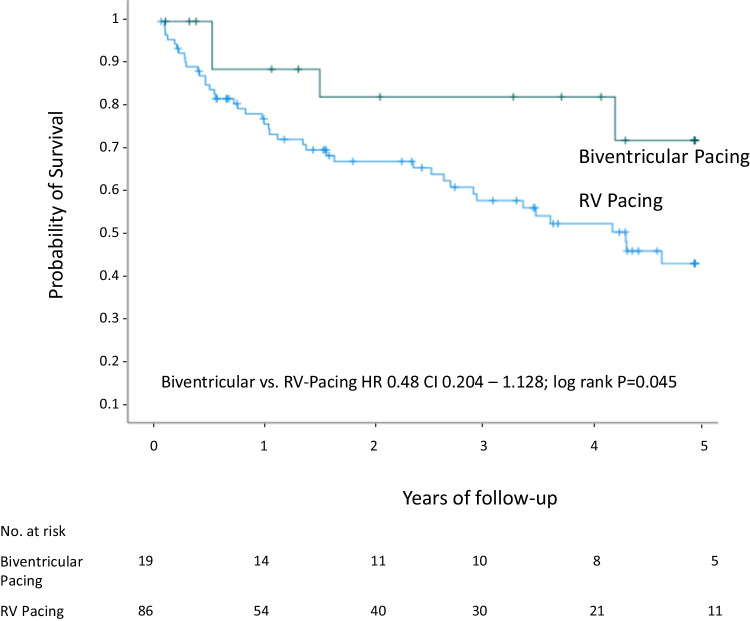
Survival after TAVR in patients with LVrEF and PMI. CI = confidence interval, CRT = cardiac resynchronization therapy, HR = hazard ratio, RV = right ventricular

In LVrEF patients, Kaplan–Meier analysis revealed that estimated all-cause mortality after a mean of 1382 ± 33 days of follow-up was significantly lower in CRT patients as compared to patients with RV pacing (21.1% vs. 48.8%; HR 0.48, CI 0.204–1.128; log rank *p* = 0.045) (Fig. 3) as well as compared to LVrEF patients not undergoing any device implantation (21.1% vs. 44.2%; HR 0.358, CI 0.133 – 0.962; log rank *p* = 0.033). After adjustment for clinical confounders, biventricular pacing at discharge remained a significant beneficial factor for 5-year patient survival in multivariate analysis (HR 0.3, CI 0.095–0.951, *p* = 0.041) (Table 3).

**Table 3:** Multivariate Cox Regression and adjustment for clinical confounders in CRT and RV pacing patients with LVrEF

Object	Hazard ratio	upper 95% CI	lower 95% CI	*p*-value
CRT pacing	0.30	0.951	0.095	**0.041**
Age	0.99	1.029	0.954	0.621
Euroscore II	1.02	1.044	0.996	0.109
Baseline creatinine	1.23	1.472	1.029	**0.023**

CI = confidence interval; CRT = cardiac resynchronization therapy;

In LVrEF patients, the CRT-group had significantly lower LVEF values prior to TAVR as compared to patients receiving RV pacing (30 ± 6.3% vs. 34.4 ± 5.9%; *p* = 0.004). QRS widths prior TAVR (129 ± 21 ms vs. 126 ± 28 ms, *p* = 0.674) and QRS widths after TAVR but before PMI (153 ± 23 ms vs. 153 ± 23 ms, *p* = 0.305) were not significantly different between CRT and RV pacing patients with LVrEF. QRS width at discharge was significantly reduced in the CRT group (145 ± 20 ms vs. 166 ± 28 ms; *p* = 0.003) and ventricular pacing burden was higher (99 ± 1% vs 79 ± 34%; *p* = 0.001) as compared to patients receiving RV pacing (Table 4). The percentage of patients receiving an ICD between CRT patients and patients receiving RV pacing was not significantly different (15.8% vs. 5.8%; *p* = 0.16). After four months (mean follow up 133 days, IQR 103–394 days), LVEF improved significantly in patients receiving CRT pacing (30 ± 6.3% to 40% (31–50%); *p* < 0.001) as well as in patients receiving RV pacing (34.4 ± 5.9% to 40% (32–45%); *p* < 0.001). Although LVEF was comparable between patients receiving CRT and RV pacing (*p* = 0.442) after four months, there was a trend towards greater LVEF change in CRT patients (14.9 ± 13.4% vs. 6.1 ± 7.9%, *p* = 0.075) (Table 4).

**Table 4:** QRS width and pacing parameters in LVrEF patients

Parameter	RV pacing	CRT	*p*-value
Patients, *n* (%)	86 (81.9)	19 (18.1)	
QRS width prior TAVR, ms	126 ± 28	129 ± 21	0.674
QRS width after TAVR prior PMI, ms	146 ± 25	153 ± 23	0.305
QRS width after PMI, ms	166 ± 28	145 ± 20	**0.003**
Pacing burden at discharge, ms	79 ± 34	99 ± 1	**0.001**
PMI with ICD, *n* (%)	5 (5.8)	3 (15.8)	0.16
LVEF prior TAVR, %	34.4 ± 5.9	30 ± 6.3	**0.004**
LVEF at discharge, %	21.5 ± 21.4	22.1 ± 16.3	0.919
LVEF four months after TAVR, %	42.3 ± 8.5	45.1 ± 12.4	0.442
LVEF Change four months after TAVR, %	6.1 ± 7.9	14.9 ± 13.4	0.075

Values are displayed as mean ± SD or frequencies (%). CRT = cardiac resynchronization therapy; LVEF = left ventricular ejection fraction; PMI = permanent pacemaker implantation, RV = right ventricle; TAVR = transcatheter aortic valve replacement

## Discussion

This study sought to analyze the impact of PMI after TAVR on overall patient survival in patients with and without reduced LVEF. The study has two major findings. First, PMI after TAVR was not associated with increased overall mortality or increased mortality in the LVrEF patient cohort. Second, in patients with LVEF ≤ 40% and PMI-indication, biventricular pacing was associated with decreased mortality as compared to right ventricular pacing.

### Risk factors for impaired survival after TAVR and impact of PMI after TAVR on patient survival

Long-term survival has become an increasingly important study endpoint as more patients undergo TAVR at younger ages [17]. Today, ESC guidelines recommend TAVR for patients ≥ 75 years whereas current ACC guidelines recommend considering clinical factors if the patient age is ≥ 65 years [18, 19] and consequently, a relevant proportion of this population has a life expectancy of 5 years and more [20].

PMI did not influence overall 5-year mortality in our patient cohort and PMI rates after TAVR were not different among patients across LVEF groups. In our cohort, the overall PMI rate was 13.8% which is comparable to other trials [20]. Several studies found adverse outcome related to PMI after 1-year follow-up duration, but these findings were not confirmed in other studies [7, 8]. In heart failure patients, RV pacing is a known factor which is associated with increased risk of mortality and hospitalization [11].

In-hospital and long-term mortality was higher for LVrEF patients as compared to patients with preserved or only mildly reduced LVEF. However, LVEF did not remain as a significant risk factor for patient mortality after multivariate cox regression analysis. This might indicate that LVEF serves as a surrogate factor but not as a true predictor of patient mortality after TAVR, especially considering that aortic stenosis might have contributed to impaired LVEF which potentially resolves after TAVR.

### What is the right pacing mode when AV block after TAVR occurs?

Individual risk for hospitalization and death after TAVR may rely on LVEF improvement and right ventricular pacing burden. Tsushima et al. found RV-pacing ≥ 30% was associated with higher rates of heart failure and death in pacemaker recipients after TAVR [21]. However, the patients in this study had preserved LVEF and did not undergo CRT implantation [21]. Predictors for high pacing burden and failure of LVEF improvement after TAVR would help to decide whether a patient needs CRT or may be treated with RV pacing alone. Additionally, there are no valid predictors for high RV pacing burden after TAVR, although the rate of recovery of AV block after TAVR is relatively low [22]. There are only limited data available on the role of CRT implantation after TAVR. According to current guidelines CRT is indicated in patients with reduced LVEF and no differentiation between patients with or without TAVR is made. In patients with LVrEF undergoing TAVR, LV function may recover. In our study, patients receiving RV pacing as well as patients receiving CRT pacing displayed significantly higher LVEF four months after TAVR. Therefore, a substantial fraction of patients with LVEF ≤ 40% at baseline had no longer an indication for CRT implantation. However, the additional effect of CRT as heart failure therapy is neglected in these patients.

It is unknown if the results of studies including patients without TAVR, like the BLOCK-HF study [11], can be transferred to this particular patient collective. In a study by Ananwattanasuk et al. electrical dyssynchrony either associated with a high burden of RV pacing or left bundle branch block was associated with increased mortality after TAVR [23].

Our study found reduced all-cause mortality associated with CRT in LVrEF patients as compared to RV pacing and highlights the potential role of biventricular pacing in these patients. It remains unclear whether patients with preserved or mildly reduced LVEF benefit from CRT-device implantation. In our cohort, only 2 of 498 patients with PMI (0.4%) and LVEF > 40% underwent CRT implantation after TAVR because current heart failure and pacing guidelines were respected for these patients. It is also not clear whether patients with reduced LVEF and a low percentage of RV pacing, either with or without procedure-induced left bundle branch block, would benefit from CRT. In our study, patients receiving CRT had a high rate of biventricular pacing which was associated with the indication-specific pacemaker programming. Due to the retrospective design of our study no definite conclusions can be drawn. Further analysis in larger scale randomized studies are needed to define the role of CRT or conduction system pacing in TAVR recipients.

We found that CRT recipients had better survival as compared to patients with reduced LV function who received RV pacing as well as to patients who did not receive any pacing modality. Reasons for this are unknown. We performed a multivariate analysis and found only one other factor (renal function) besides CRT which was associated with different patient survival. A relatively high amount of patients with left bundle branch block who did not receive CRT implantation directly after TAVR (due to missing high-degree AV block) may play a role. Additionally, patients who received a CRT device may benefit from concomitant ICD therapy. These observations need further investigation in future studies on larger patient cohorts.

QRS width in the CRT group was 145 ± 20 ms which might have been optimized during follow up. However, it has been shown that QRS width < 150 ms during biventricular pacing is associated with reduced risk of heart failure and death [22, 23].

### Limitations

There are several limitations with this study. At first, we could not distinguish between cardiovascular and non-cardiovascular deaths. Furthermore, there are no information regarding the hospitalization rate which serves as a relevant endpoint in most other trials. Additionally, the number of patients with LVrEF and PMI was relatively small compared to other trials.

This is a retrospective study and patients were not randomized. We do not have any further information regarding the cause of death. The number of patients who received PPI and CRT was relatively small. Albeit no statistically different amount of patients who received ICD therapy among patients with RV pacing and CRT (5 vs. 15%), this numerical difference may have played a role with regards to patient survival. Patients were treated by CRT or RV pacing after individual decision for each patient based on the preference of treating physicians and patients taking potential LV recovery as well as patient comorbidities into account. Multivariate analysis was performed to eliminate potential confounders. Nevertheless, there might have been several clinical reasons that have contributed to the decision for or against pacemaker or CRT. Adjustment for these reasons is difficult and might not be part of multivariate analyses.

## Conclusion

In patients undergoing TAVR with preserved LVEF, pacemaker implantation was not associated with increased mortality. In patients with reduced LVEF, CRT implantation was associated with reduced all-cause mortality as compared to RV pacing. Randomized trials are needed to compare RV pacing with biventricular pacing in LVrEF patients after TAVR.

## Supplementary Information

Below is the link to the electronic supplementary material.

Supplementary file1 (DOCX 32 KB)

## Data Availability

Original data are available on reasonable request.

## Abbreviations

CRT: Cardiac resynchronization therapy
HfrEF: Heart failure with reduced ejection fraction
LVEF: Left ventricular ejection fraction
LVmrEF: Left ventricle with mildly reduced ejection fraction
LVpEF: Left ventricle with preserved ejection fraction
LvrEF: Left ventricle with reduced ejection fraction
PMI: Pacemaker implantation
RBBB: Right bundle branch block
TAVR: Transcatheter aortic valve replacement

## References

[1] Auffret V, Puri R, Urena M, Chamandi C, Rodriguez-Gabella T, Philippon F, Rodés-Cabau J (2017) Conduction disturbances after transcatheter aortic valve replacement: current status and future perspectives. Circulation 136(11):1049–1069. 10.1161/CIRCULATIONAHA.117.028352 28893961 10.1161/CIRCULATIONAHA.117.028352

[2] Glikson M, Nielsen JC, Kronborg MB, Michowitz Y, Auricchio A, Barbash IM, Barrabés JA, Boriani G, Braunschweig F, Brignole M, Burri H, Coats AJS, Deharo JC, Delgado V, Diller GP, Israel CW, Keren A, Knops RE, Kotecha D, Leclercq C, Merkely B, Starck C, Thylén I, Tolosana JM, Leyva F, Linde C, Abdelhamid M, Aboyans V, Arbelo E, Asteggiano R, Barón-Esquivias G, Bauersachs J, Biffi M, Birgersdotter-Green U, Bongiorni MG, Borger MA, Čelutkienė J, Cikes M, Daubert JC, Drossart I, Ellenbogen K, Elliott PM, Fabritz L, Falk V, Fauchier L, Fernández-Avilés F, Foldager D, Gadler F, De Vinuesa PGG, Gorenek B, Guerra JM, Hermann Haugaa K, Hendriks J, Kahan T, Katus HA, Konradi A, Koskinas KC, Law H, Lewis BS, Linker NJ, Løchen ML, Lumens J, Mascherbauer J, Mullens W, Nagy KV, Prescott E, Raatikainen P, Rakisheva A, Reichlin T, Ricci RP, Shlyakhto E, Sitges M, Sousa-Uva M, Sutton R, Suwalski P, Svendsen JH, Touyz RM, Van Gelder IC, Vernooy K, Waltenberger J, Whinnett Z, Witte KK (2022) 2021 ESC Guidelines on cardiac pacing and cardiac resynchronization therapy. Europace 24(1):71–164. 10.1093/europace/euab232. (**Erratum in: Europace. 2022 Mar 07**) 34455427 10.1093/europace/euab232 PMC13179788

[3] van Rosendael PJ, Delgado V, Bax JJ (2018) Pacemaker implantation rate after transcatheter aortic valve implantation with early and new-generation devices: a systematic review. Eur Heart J 39(21):2003–2013. 10.1093/eurheartj/ehx785 29420704 10.1093/eurheartj/ehx785

[4] Fujita B, Schmidt T, Bleiziffer S, Bauer T, Beckmann A, Bekeredjian R, Möllmann H, Walther T, Landwehr S, Hamm C, Beyersdorf F, Katus HA, Harringer W, Ensminger S, Frerker C, GARY Executive Board (2020) Impact of new pacemaker implantation following surgical and transcatheter aortic valve replacement on 1-year outcome. Eur J Cardiothorac Surg. 57(1):151–159. 10.1093/ejcts/ezz168 31199470 10.1093/ejcts/ezz168

[5] Fadahunsi OO, Olowoyeye A, Ukaigwe A, Li Z, Vora AN, Vemulapalli S, Elgin E, Donato A (2016) Incidence, predictors, and outcomes of permanent pacemaker implantation following transcatheter aortic valve replacement: analysis from the U.S. society of thoracic surgeons/American college of cardiology TVT registry. JACC Cardiovasc Interv. 9(21):2189–2199. 10.1016/j.jcin.2016.07.026 27832844 10.1016/j.jcin.2016.07.026

[6] Chamandi C, Barbanti M, Munoz-Garcia A, Latib A, Nombela-Franco L, Gutiérrez-Ibanez E, Veiga-Fernandez G, Cheema AN, Cruz-Gonzalez I, Serra V, Tamburino C, Mangieri A, Colombo A, Jiménez-Quevedo P, Elizaga J, Laughlin G, Lee DH, Garcia Del Blanco B, Rodriguez-Gabella T, Marsal JR, Côté M, Philippon F, Rodés-Cabau J (2018) Long-term outcomes in patients with new permanent pacemaker implantation following transcatheter aortic valve replacement. JACC Cardiovasc Interv 11(3):301–310. 10.1016/j.jcin.2017.10.032 29413244 10.1016/j.jcin.2017.10.032

[7] Rück A, Saleh N, Glaser N (2021) Outcomes following permanent pacemaker implantation after transcatheter aortic valve replacement: SWEDEHEART observational study. JACC Cardiovasc Interv 14(19):2173–2181. 10.1016/j.jcin.2021.07.043 34620397 10.1016/j.jcin.2021.07.043

[8] Natanzon SS, Fardman A, Koren-Morag N, Fefer P, Maor E, Guetta V, Segev A, Barbash I, Nof E, Beinart R (2022) Pacing burden and clinical outcomes after transcatheter aortic valve replacement-A real-world registry report. Heart Rhythm 19(9):1508–1515. 10.1016/j.hrthm.2022.04.030 35525423 10.1016/j.hrthm.2022.04.030

[9] Sharma AD, Rizo-Patron C, Hallstrom AP, O’Neill GP, Rothbart S, Martins JB, Roelke M, Steinberg JS, Greene HL, DAVID Investigators (2005) Percent right ventricular pacing predicts outcomes in the DAVID trial. Heart Rhythm 2(8):830–4. 10.1016/j.hrthm.2005.05.015 16051118 10.1016/j.hrthm.2005.05.015

[10] Curtis AB, Worley SJ, Adamson PB, Chung ES, Niazi I, Sherfesee L, Shinn T, Sutton MS, Biventricular versus Right Ventricular Pacing in Heart Failure Patients with Atrioventricular Block (BLOCK HF) Trial Investigators (2013) Biventricular pacing for atrioventricular block and systolic dysfunction. N Engl J Med. 368(17):1585–93. 10.1056/NEJMoa1210356 23614585 10.1056/NEJMoa1210356

[11] Kusumoto FM, Schoenfeld MH, Barrett C et al (2019) 2018 ACC/AHA/HRS guideline on the evaluation and management of patients with bradycardia and cardiac conduction delay: a report of the American college of cardiology/American heart association task force on clinical practice guidelines and the heart rhythm society. J Am Coll Cardiol 74(7):e51–e156. 10.1016/j.jacc.2018.10.044 30412709 10.1016/j.jacc.2018.10.044

[12] Baron SJ, Arnold SV, Herrmann HC, Holmes DR Jr, Szeto WY, Allen KB, Chhatriwalla AK, Vemulapali S, O’Brien S, Dai D, Cohen DJ (2016) Impact of ejection fraction and aortic valve gradient on outcomes of transcatheter aortic valve replacement. J Am Coll Cardiol 67(20):2349–2358. 10.1016/j.jacc.2016.03.514 27199058 10.1016/j.jacc.2016.03.514 PMC5372353

[13] Carreras ET, Kaneko T, Ramirez-Del Val F, Pelletier MP, Sobieszczyk PS, Bhatt DL, Shah PB (2018) Impact of flow, gradient, and left ventricular function on outcomes after transcatheter aortic valve replacement. Catheter Cardiovasc Interv 91(4):798–805. 10.1002/ccd.27347 28988432 10.1002/ccd.27347 PMC5849510

[14] Elmariah S, Palacios IF, McAndrew T, Hueter I, Inglessis I, Baker JN, Kodali S, Leon MB, Svensson L, Pibarot P, Douglas PS, Fearon WF, Kirtane AJ, Maniar HS, Passeri JJ, PARTNER Investigators (2013) Outcomes of transcatheter and surgical aortic valve replacement in high-risk patients with aortic stenosis and left ventricular dysfunction: results from the Placement of Aortic Transcatheter Valves (PARTNER) trial (cohort A). Circ Cardiovasc Interv 6(6):604–14. 10.1161/CIRCINTERVENTIONS.113.000650 24221391 10.1161/CIRCINTERVENTIONS.113.000650

[15] Clavel MA, Webb JG, Rodés-Cabau J, Masson JB, Dumont E, De Larochellière R, Doyle D, Bergeron S, Baumgartner H, Burwash IG, Dumesnil JG, Mundigler G, Moss R, Kempny A, Bagur R, Bergler-Klein J, Gurvitch R, Mathieu P, Pibarot P (2010) Comparison between transcatheter and surgical prosthetic valve implantation in patients with severe aortic stenosis and reduced left ventricular ejection fraction. Circulation 122(19):1928–1936. 10.1161/CIRCULATIONAHA.109.929893 20975002 10.1161/CIRCULATIONAHA.109.929893

[16] Maes F, Lerakis S, Barbosa Ribeiro H, Gilard M, Cavalcante JL, Makkar R, Herrmann HC, Windecker S, Enriquez-Sarano M, Cheema AN, Nombela-Franco L, Amat-Santos I, Muñoz-García AJ, Garcia Del Blanco B, Zajarias A, Lisko JC, Hayek S, Babaliaros V, Le Ven F, Gleason TG, Chakravarty T, Szeto W, Clavel MA, de Agustin A, Serra V, Schindler JT, Dahou A, Salah-Annabi M, Pelletier-Beaumont E, Côté M, Puri R, Pibarot P, Rodés-Cabau J (2019) Outcomes from transcatheter aortic valve replacement in patients with low-flow, low-gradient aortic stenosis and left ventricular ejection fraction less than 30%: a substudy from the TOPAS-TAVI registry. JAMA Cardiol 4(1):64–70. 10.1001/jamacardio.2018.4320 30566185 10.1001/jamacardio.2018.4320 PMC6439680

[17] Sedrakyan A, Dhruva SS, Sun T, Mao J, Gaudino MFL, Redberg RF (2018) Trends in use of transcatheter aortic valve replacement by age. JAMA 320(6):598–600. 10.1001/jama.2018.9938 30039166 10.1001/jama.2018.9938 PMC6584325

[18] Vahanian A, Beyersdorf F, Praz F, Milojevic M, Baldus S, Bauersachs J, Capodanno D, Conradi L, De Bonis M, De Paulis R, Delgado V, Freemantle N, Gilard M, Haugaa KH, Jeppsson A, Jüni P, Pierard L, Prendergast BD, Sádaba JR, Tribouilloy C, Wojakowski W, ESC/EACTS Scientific Document Group (2022) 2021 ESC/EACTS guidelines for the management of valvular heart disease. Eur Heart J 43(7):561–632. 10.1093/eurheartj/ehab395 34453165 10.1093/eurheartj/ehab395

[19] Sundt TM, Jneid H (2021) Guideline update on indications for transcatheter aortic valve implantation based on the 2020 American college of cardiology/American heart association guidelines for management of valvular heart disease. JAMA Cardiol 6(9):1088–1089. 10.1001/jamacardio.2021.2534 34287627 10.1001/jamacardio.2021.2534

[20] Makkar RR, Thourani VH, Mack MJ, Kodali SK, Kapadia S, Webb JG, Yoon SH, Trento A, Svensson LG, Herrmann HC, Szeto WY, Miller DC, Satler L, Cohen DJ, Dewey TM, Babaliaros V, Williams MR, Kereiakes DJ, Zajarias A, Greason KL, Whisenant BK, Hodson RW, Brown DL, Fearon WF, Russo MJ, Pibarot P, Hahn RT, Jaber WA, Rogers E, Xu K, Wheeler J, Alu MC, Smith CR, Leon MB, PARTNER 2 Investigators (2020) Five-year outcomes of transcatheter or surgical aortic-valve replacement. N Engl J Med 382(9):799–809. 10.1056/NEJMoa1910555 31995682 10.1056/NEJMoa1910555

[21] Tsushima T, Al-Kindi S, Palma Dallan LA, Fares A, Yoon SH, Wheat HL, Attizzani GF, Baeza CR, Pelletier MP, Arruda MS, Mackall JA, Thal SG (2023) Clinical impact of right ventricular pacing burden in patients with post-transcatheter aortic valve replacement permanent pacemaker implantation. Europace 25(4):1441–1450. 10.1093/europace/euad025 36794441 10.1093/europace/euad025 PMC10105841

[22] Mazzella AJ, Arora S, Hendrickson MJ, Sanders M, Vavalle JP, Gehi AK (2021) Evaluation and management of heart block after transcatheter aortic valve replacement. Card Fail Rev 7:e12. 10.15420/cfr.2021.05 34386266 10.15420/cfr.2021.05 PMC8353545

[23] Ananwattanasuk T, Atreya AR, Teerawongsakul P, Ghannam M, Lathkar-Pradhan S, Latchamsetty R, Jame S, Patel HJ, Grossman PM, Oral H, Jongnarangsin K (2023) Outcomes in patients with electrocardiographic left ventricular dyssynchrony following transcatheter aortic valve replacement. Heart Rhythm 20(1):22–28. 10.1016/j.hrthm.2022.08.001 35948202 10.1016/j.hrthm.2022.08.001

