# Supplementary material for: Mortality after cardiac resynchronization therapy or right ventricular pacing in transcatheter aortic valve replacement recipients
Source: Clin Res Cardiol. 2024 May 2;115(4):536–45. doi: 10.1007/s00392-024-02450-1 (PMC13013165; doi:10.1007/s00392-024-02450-1)
Supplement: Supplementary file 1 — Supplementary file1 (DOCX 32 KB) [file 392_2024_2450_MOESM1_ESM.docx]

**Supplemental table 1: Echocardiographic parameters**

| **Parameter** | **LVrEF** | **LVmrEF** | **LVpEF** | **p-value** |
| --- | --- | --- | --- | --- |
| LVEF prior TAVR, % | 34.0±6.3 | 45.2±1.5 | 55.9±4.3 | **<0.001** |
| AVA prior TAVR, cm² | 0.71±0.22 | 0.71±0.18 | 0.75±0.21 | **<0.001** |
| MPG prior TAVRm mmHg | 37.9±15.4 | 41.4±16.8 | 47.1±22.1 | **<0.001** |
| MPG after TAVR, mmHg | 8.8±4.7 | 9.6±5.2 | 10.2±5.3 | **<0.001** |
| LVEF post TAVR, % | 38.8±9.9 | 47.6±6.3 | 55.3±11.8 | **<0.001** |
| EOA, cm² | 1.74±0.47 | 1.77±0.44 | 1.81±0.46 | **0.010** |
| Non AR post TAVR, n (%) | 234 (33.5) | 123 (40.3) | 1208 (35.7) | 0.805 |
| AR I° post TAVR, n (%) | 172 (24.6) | 78 (25.6) | 924 (27.3) |  |
| AR II° post TAVR, n (%) | 7 (1.0) | 2 (0.7) | 33 (1.0) |  |
| AR III° post TAVR, n (%) | 0 | 0 | 1 (0.03) |  |

Values are displayed as mean±SD or frequencies (%). AR=aortic regurgitation; AVA=aortic valve area; EOA=effective orifice area; LVEF=left ventricular ejection fraction; MPG=mean pressure gradient; TAVR=transcatheter aortic valve replacement

**Supplemental table 2. Multivariate Cox Regression analysis on patient survival after TAVR**

| **Parameter** | **Hazard ratio** | **upper 95,0% CI** | **lower 95% CI** | **p-value** |
| --- | --- | --- | --- | --- |
| Age, years | 1.090 | 1.034 | 1.149 | **0.001** |
| BMI, kg / m² | 0.997 | 0.947 | 1.049 | 0.905 |
| Sex female, n (%) | 0.828 | 0.487 | 1.408 | 0.486 |
| LVEF, % | 1.004 | 0.962 | 1.048 | 0.847 |
| MPG prior TAVR, mmHg | 0.996 | 0.984 | 1.007 | 0.445 |
| Euroscore II | 1.032 | 0,983 | 1.084 | 0.199 |
| NYHA II | 0.511 | 0.064 | 4.102 | 0.528 |
| NYHA III | 0.772 | 0.099 | 5.993 | 0.804 |
| NYHA IV | 1.789 | 0.170 | 18.786 | 0.628 |
| Diabetes mellitus | 1.668 | 0.960 | 2.898 | 0.070 |
| Hypertension | 0.512 | 0.206 | 1.273 | 0.150 |
| CAD | 0.848 | 0.494 | 1.455 | 0.549 |
| Creatinine | 1.335 | 1.184 | 1.504 | **<0.001** |
| Stroke/TIA | 0.935 | 0,445 | 1.966 | 0.860 |
| MPG after TAVR | 0.966 | 0.921 | 1.013 | 0.155 |

BMI=body mass index; CAD=coronary artery disease; CI=confidence interval; LVEF=left ventricular ejection fraction; MPG=mean pressure gradient; NYHA=New York Heart Association; TIA=transient ischemic attack; TAVR=transcatheter aortic valve replacement.

**Supplemental table 3. Cox-regression analysis on impact of PMI on survival after TAVR.**

| **Subgroup** | **Estimated rate of death in patients without PMI vs. with PPI at 5 years** | **HR (95% CI)** | **log rank p-value** |
| --- | --- | --- | --- |
| Overall | 48.6% vs. 48.5% | 0.977 (0.906 – 1.055) | 0.552 |
| LVpEF | 45.1% vs. 43.9% | 0.980 (0.893 – 1.077) | 0.613 |
| LVmrEF | 52.8% vs. 76.6% | 0.879 (0.692 – 1.118) | 0.294 |
| LVrEF | 73.2% vs. 67.5% | 1.070 (0.914 – 1.251) | 0.399 |

Hazard ratio is displayed with 95% confidence interval in brackets and p-value. CI=confidence interval; HR=hazard ratio; LVmrEF=left ventricle with mildly reduced ejection fraction; LVpEF=left ventricle with preserved ejection fraction; LVrEF=left ventricle with reduced ejection fraction, PMI=pacemaker implantation

| **Parameter** | **All** | **LVrEF** | **LVmrEF** | **LVpEF** | **P-value**  **LVrEF vs. LVmrEF** | **P-value**  **LVmrEF vs. LVpEF** | **P-value**  **LVrEF vs. LVpEF** |
| --- | --- | --- | --- | --- | --- | --- | --- |
| n | 4385 | 698 | 305 | 3382 |  |  |  |
| Male, n (%) | 2055 (46.9) | 433 (62.0) | 175 (57.4) | 1447 (42.8) | 0.182 | **<0.001** | **<0.001** |
| Age, years | 81.4±6.1 | 80.8±7.3 | 81±6 | 81.6±5.8 | 0.724 | 0.151 | 0.139 |
| BMI, kg / m² | 27.3±5.2 | 26.7±5.2 | 27.5±5.5 | 27.4±5.1 | **<0.001** | 0.84 | **0.022** |
| Euroscore II | 5.9±6.5 | 12.9±11.2 | 7.3±6.3 | 4.7±4.3 | **<0.001** | **<0.001** | **<0.001** |
| STS-Score | 5.7±4.6 | 7.9±7 | 6.2±4.5 | 5.2±3.7 | **<0.001** | **<0.001** | **<0.001** |
| LVEF, % | 51.7±9.4 | 34±6.3 | 45.2±1.5 | 55.9±4.3 | **<0.001** | **<0.001** | **<0.001** |
| Creatinine, mg/dl | 1.5±1.3 | 1.5±1.1 | 1.4±0.9 | 1.2±0.7 | **0.015** | 0.59 | **0.019** |
| NYHA I, n (%) | 117 (2.7) | 9 (1.3) | 4 (1.3) | 104 (3.1) | **0.026** | **<0.001** | **<0.001** |
| NYHA II, n (%) | 1299 (29.6) | 131 (18.8) | 73 (23.9) | 1095 (32.4) |  |  |  |
| NYHA III, n (%) | 2707 (61.7) | 449 (64.3) | 200 (65.6) | 2058 (60.9) |  |  |  |
| NYHA IV, n (%) | 262(6.0) | 109 (15.6) | 28 (9.2) | 125 (3.7) |  |  |  |
| CVD, n (%) | 642 (14.6) | 86 (12.3) | 42 (13.8) | 514 (15.2) | 0.538 | 0.559 | 0.053 |
| PAD, n (%) | 537 (12.2) | 116 (16.6) | 46 (15.1) | 375 (11.1) | 0.577 | 0.39 | **<0.001** |
| Hypertension, n (%) | 3988 (91.1) | 609 (87.2) | 287 (94.1) | 3097 (91.57) | **0.006** | 0.303 | **0.002** |
| Diabetes mellitus, n (%) | 1289 (29.5) | 250 (36.3) | 117 (38.4) | 927 (27.4) | 0.963 | **0.004** | **<0.001** |
| Hyperlipidemia, n (%) | 3395 (80.3) | 525 (75.2) | 249 (81.6) | 2778 (82.1) | 0.12 | 0.436 | **<0.001** |
| No CAD, n (%) | 1861 (42.2) | 241 (34.5) | 103 (33.8) | 1517 (44.9) | 0.062 | **0.002** | **<0.001** |
| CAD, 1 vessel, n (%) | 887 (20.2) | 133 (19.1) | 72 (23.6) | 682 (20.2) |  |  |  |
| CAD, 2 vessel, n (%) | 635 (14.5) | 105 (15.0) | 56 (18.4) | 474 (14.0) |  |  |  |
| CAD, 3 vessel, n (%) | 1002 (22.9) | 219 (34.5) | 74 (24.3) | 709 (21.0) |  |  |  |
| Prior stent implantation, n (%) | 1449 (33.9) | 271 (38.8) | 122 (40) | 1056 (31.2) | 0.726 | **0.002** | **<0.001** |
| Atrial Fibrillation, n (%) | 1618 (36.9) | 326 (46.7) | 148 (48.5) | 1135 (33.6) | 0.17 | **<0.001** | **<0.001** |
| Prior Stroke / TIA, n (%) | 3759 (85.7) | 580 (83.1) | 248 (81.3) | 2931 (86.7) | 0.527 | **0.012** | **0.016** |
| Dialysis , n (%) | 127 (2.8) | 48 (6.9) | 11 (3.6) | 68 (2.0) | 0.125 | 0.156 | **<0.001** |
| COPD, n (%) | 856 (19.5) | 154 (22.1) | 60 (19.7) | 642 (19.0) | 0.687 | 0.254 | 0.077 |

**Supplemental Table 4 Baseline Characteristics**

Values are displayed as mean±SD or frequencies (%). BMI=body mass index; CAD=coronary artery disease; COPD=chronic obstructive pulmonary disease; CVD=cervical vascular disease; NYHA=New York Heart Association; PAD=peripheral artery disease; STS=Society of Thoracic Surgery; TIA=transient ischemic attack

**Supplemental Table 5 Baseline parameters in LVrEF patients receiving RV, CRT or no pacing**

| **Parameter** | **RV pacing** | **CRT** | **No pacing** | **P-value** |
| --- | --- | --- | --- | --- |
| Patients, n (%) | 86 (81.9) | 19 (18.1) | 593 (84.9) |  |
| Male Gender, n (%) | 66 (76.7) | 13 (68.4) | 354 (59.7) | 0.098 |
| BMI, kg / m² | 26.6±5.0 | 29.6±6.4 | 26.6±5.2 | 0.102 |
| EuroScore II | 5.0 (2.8-12.6) | 7.4 (5.8-12.6) | 6.9 (4.2-11.0) | 0.165 |
| Creatinine, mg/dl | 1.7±1.4 | 1.3±0.5 | 1.5±1.1 | 0.406 |
| Diabetes Mellitus, n (%) | 44 (51.2) | 10 (52.6) | 197 (33.2) | **0.002** |
| Previous stroke, n (%) | 69 (80.2) | 16 (84.2) | 495 (83.5) | 0.749 |
| Atrial fibrillation, n (%) | 41 (47.7) | 8 (42.1) | 277 (46.7) | 0.908 |
| Dialysis, n (%) | 9 (10.5) | 0 (0) | 39 (6.6) | 0.151 |
| LVEF prior TAVR, (%) | 34.4±5.9 | 30±6.3 | 34.4±6.4 | **0.01** |
| LVEF at discharge, (%) | 30 (26-39) | 40 (31-50) | 40 (32-45) | **0.017** |
| NYHA, n (%)  I, n (%)  II, n (%)  III, n (%)  IV, n (%) | 0 (0)  14 (16.3)  58 (67.4)  14 (16.3) | 0 (0)  3 (15.8)  12 (63.1)  4 (21.1) | 9 (0.2)  114 (19.2)  379 (63.9)  91 (15.3) | 0.858 |

Values are displayed as mean±SD or frequencies (%). CRT=cardiac resynchronization therapy; NYHA=New York Heart Association; LVEF=left ventricular ejection fraction; RV=right ventricle;
